# Supplementary material for: Mechanical plasticity of collagen directs branch elongation in human mammary gland organoids
Source: Nat Commun. 2021 May 12;12:2759. doi: 10.1038/s41467-021-22988-2 (PMC8115695; doi:10.1038/s41467-021-22988-2)
Supplement: Supplementary file 1 — Supplementary Information [file 41467_2021_22988_MOESM1_ESM.pdf]

Supplementary information

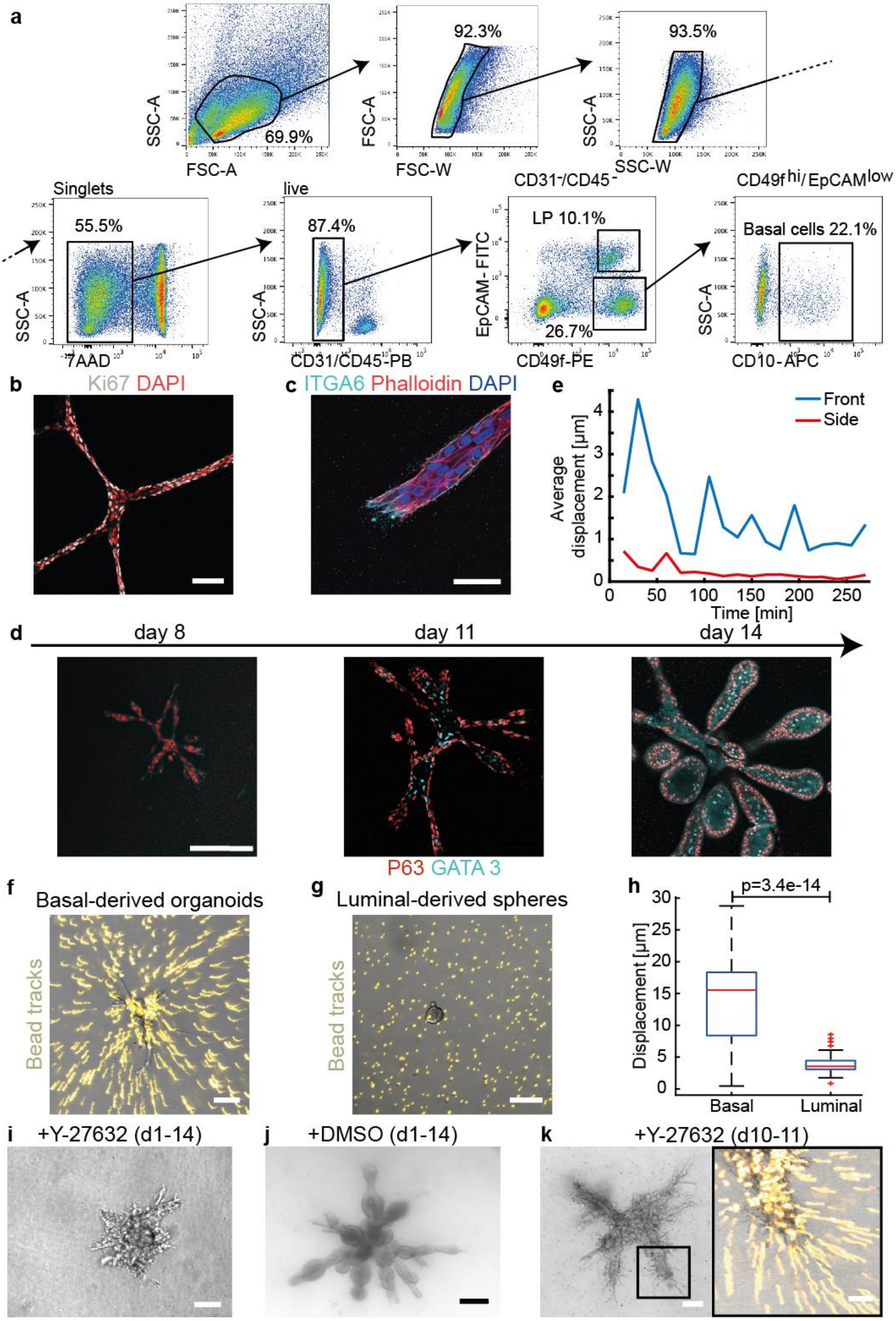

Fig. S1: a FACS gating strategy to sort basal mammary epithelial cells (EpCAM$^-$/CD49f^{hi}/CD10$^+$) from human reduction mammoplasties displayed in pseudocolor plots and histograms. Forward scatter (FSC) and Sideward scatter (SSC) were used for exclusion of cell debris and doublets, dead cells (7AAD$^-$ = live), hematopoietic (CD45$^+$), and endothelial cells (CD31$^+$) were excluded. By using the markers EpCAM, CD49f and CD10, the basal population (EpCAM$^-$/CD49f^{hi}/CD10$^+$) was isolated. The colors in the pseudocolor plots refer to the density of the cells relative to one another. Blue and green thereby indicate areas of low cell density, yellow is mid-range density, orange and red areas are areas of high cell density. **b** Immunofluorescence staining of Ki67 shows proliferative cells throughout the whole organoid during branch elongation phase. **c** Immunofluorescence staining of Integrin alpha 6 and phalloidin shows invasive elongation behavior of the branches. **d** Immunofluorescence staining of p63 and GATA3 reveals bilayered architecture during the different developmental stages. **e** Representative average bead displacement in the front (blue) and at the sides (red) of a branch. **f**, **g** Representative deformation field of (**f**) basal cells at day 7 over a time course of 45 hrs and (**g**) luminal cells at day 7 over a time period of 19 hrs. **h** Bead displacements for basal and luminal structures, both over a time period of 12 hrs. Box plots indicate median (red line), 25th,75th percentile (blue box) and 5th and 95th percentile (whiskers) as well as outliers (single points). **i, j** Characteristic morphology of organoids after continuous treatment with (**i**) ROCK inhibitor Y-27632 (10µM) and (**j**) related DMSO control. **k** Left: Characteristic morphology of organoids after onetime treatment with Y-27632 during their branch elongation phase at day 10. Right: Alternated deformation field after treatment with Y-27632. Scale bars, 100 µm (**b**,**d**-**k**), 50 µm (**c,** zoom **k**). Organoids were derived from three biologically independent donors (Table S1). In box plots, the center line indicates the median, box boundaries indicate the 25th and the 75th percentiles. P values are from a two-tailed Mann-Whitney test and provided in Table S5. Source data are provided as a Source Data file.

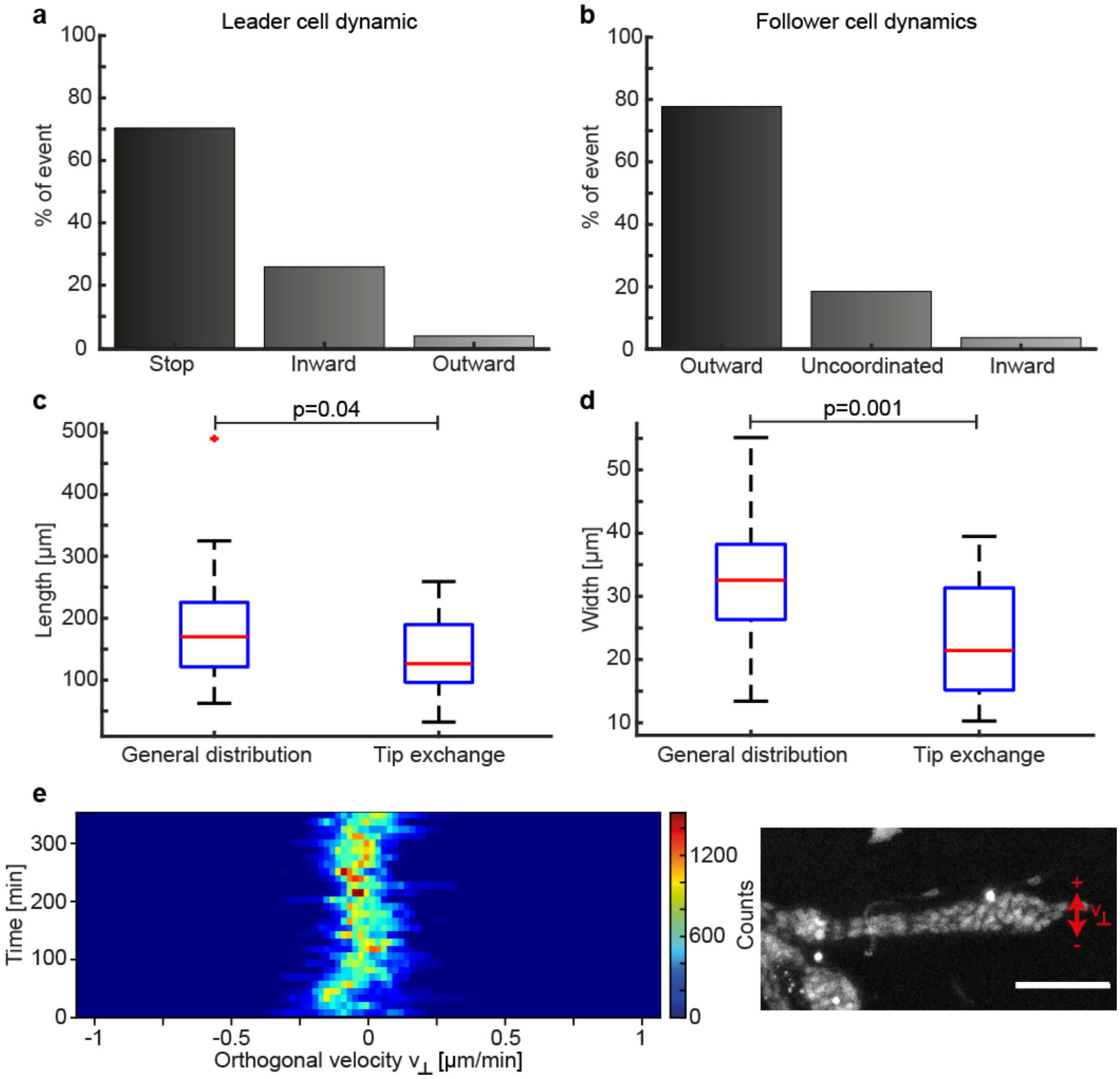

Fig. S2 a, **b** Distribution of (**a)** leader cell dynamics and (**b**) follower cell dynamics prior to an exchange (n=24). **c** Quantification of branch length of organoids in their elongation phase and branch length of branches in which a tip exchange was observed ($n_{General}$ = 45 organoids, $n_{Exchange}$ = 24 organoids). **d** Quantification of branch width of organoids in their elongation phase and branch width of branches in which a tip exchange was observed (n_{Gneral} = 45 organoids, $n_{Exchange}$ = 24 organoids). Box plots indicate median (red line), 25th,75th percentile (blue box) and 5th and 95th percentile (whiskers) as well as outliers (single points). **e** Orthogonal cell velocity $v_{\perp}$ within a representative branch throughout the elongation. Scale bar, 100 µm (**e**). Organoids were derived from three biologically independent donors (Table S1). In box plots, the center line indicates the median, box boundaries indicate the 25th and the 75th percentiles. P values are from a two-tailed Mann-Whitney test and provided in Table S5, *P < 0.5, **P < 0.01. Source data are provided as a Source Data file.

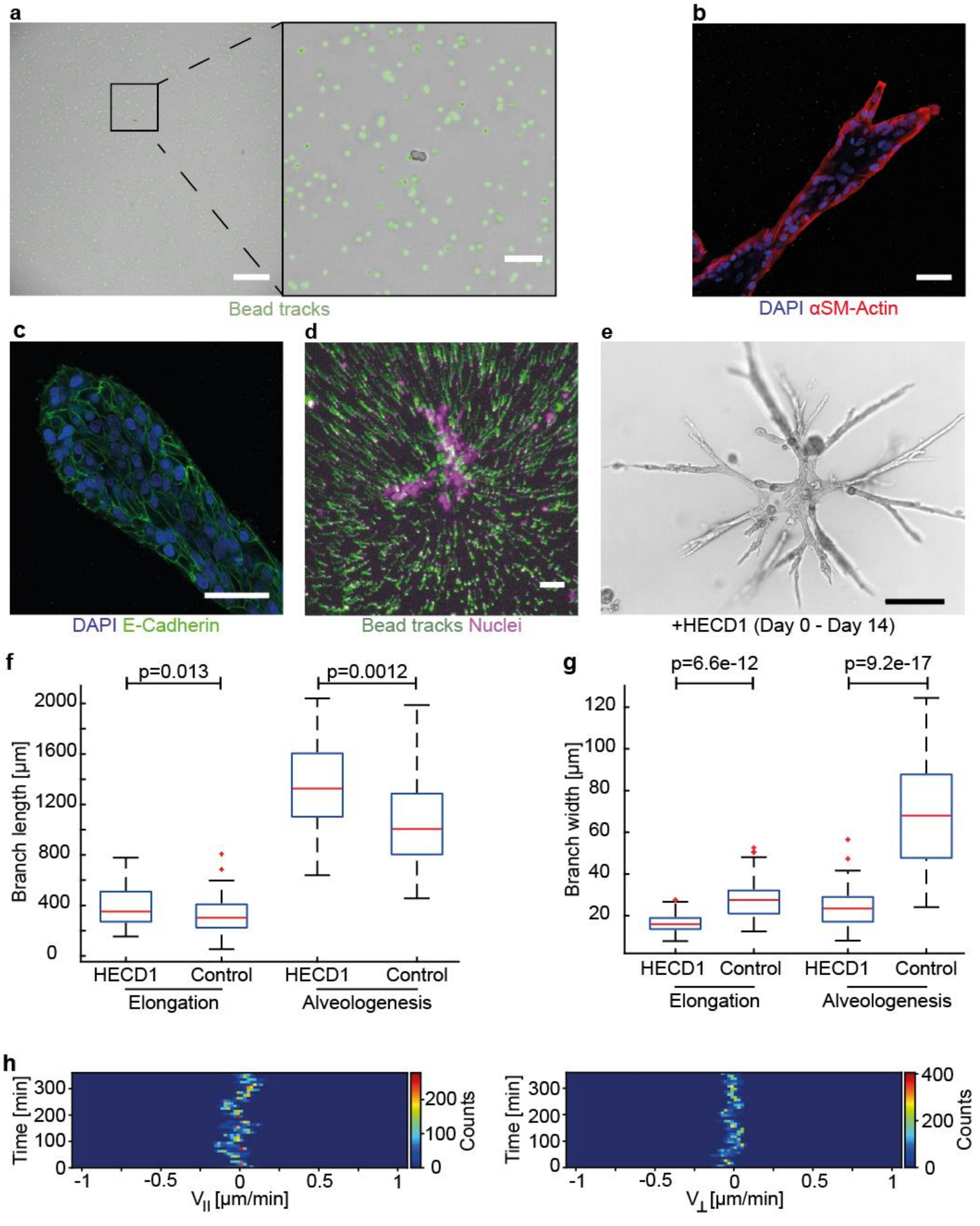

Fig. S3: a Deformation field of a single basal cell over a time period of 21 hrs. **b, c** Immunostaining against (**b**) smooth muscle actin and (**c**) E-cadherin. Cells are visualized using DAPI. **d** Time projection to visualize the initial displacement field prior to Cytochalasin D treatment. Tracks are visualized in green, nuclei and final position of the beads in magenta. **e** Representative organoid morphology after treatment of HECD1 throughout the whole organoid culture. **f, g** Branches of organoids treated with HECD1 grow (**f**) longer, but (**g**) thinner. Box plots indicate median (red line), 25th,75th percentile (blue box) and 5th and 95th percentile (whiskers) as well as outliers (single points). **h** Velocity distribution of the parallel $v_{||}$ and orthogonal $v_{\perp}$ proportion within branches of organoids treated with HECD1. Scale bars, 200 µm (**a**, **e**) 50 m (zoom **a**, **b**-**d**). Organoids were derived from three biologically independent donors (Table S1). In box plots, the center line indicates the median, box boundaries indicate the 25th

and the 75th percentiles. P values are from a two-tailed Mann-Whitney test and provided in Table S5. Source data are provided as a Source Data file.

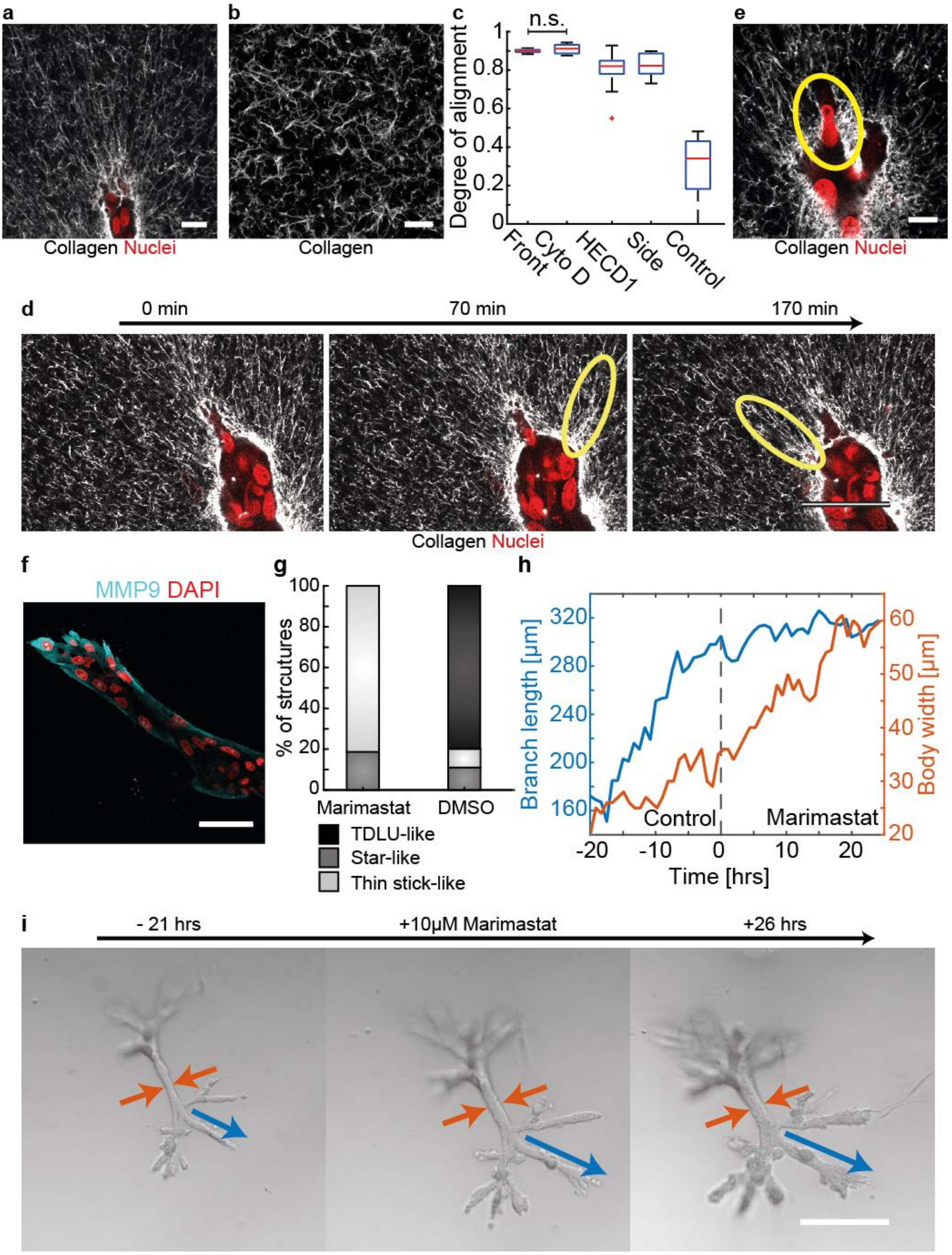

Fig. S4: a Collagen fibers are highly aligned in front of invading branches. **b** Collagen structure far away from organoids shows no preferred orientation. **c** Degree of collagen fiber alignment measured for different regions and inhibitor treatments. For the inhibitor treatments the alignment was calculated in front of the branches. After treatment with Cytochalasin D no significant difference in the fiber alignment compared to the front of untreated organoids can be detected. All other treatments lead to a significant difference in fiber alignment. Box plots indicate median (red line), 25th,75th percentile (blue box) and 5th and 95th percentile (whiskers) as well as outliers (single points). **d** Time course of

alternating fiber alignment of the invading branch (yellow circle). **e** Cells at the leading edge squeeze through holes in the densified collagen by deformation of their nucleus. **f** Immunofluorescence staining of MMP9 shows localized expression in the leading cells. **g** The formation of TDLU-like structures is inhibited, when using 10µM Marimastat throughout the whole organoid culture. **h** By inhibiting MMPs, branch elongation is hampered (blue line), while the body width is still increasing (orange line). **i** Experimental procedure and representative brightfield pictures of an organoid treated with 10µM MMP inhibitor Marimastat. Organoid body width increases over time (red arrows), while branch elongation is hampered after treatment (blue arrows). Scale bars, 50 µm (**a**, **b**, **d**, **f**), 10 µm (**e**) 300 µm (**i**). Organoids were derived from three biologically independent donors (Table S1). P values are from a two-tailed Mann-Whitney test and provided in Table S5. Source data are provided as a Source Data file.

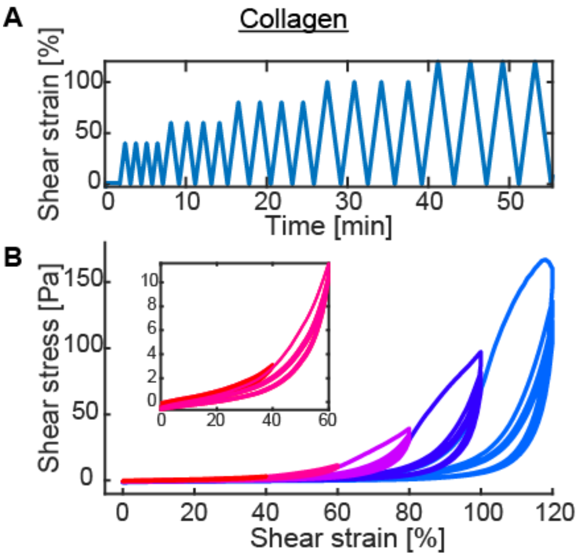

Fig. S5: Shear rheology of Collagen. a Experimental protocol for cyclic strain with increasing amplitude. **b** Collagen networks show non-linear response upon shear stress. During cyclic shearing the Mullins-softening can be observed. Source data are provided as a Source Data file.

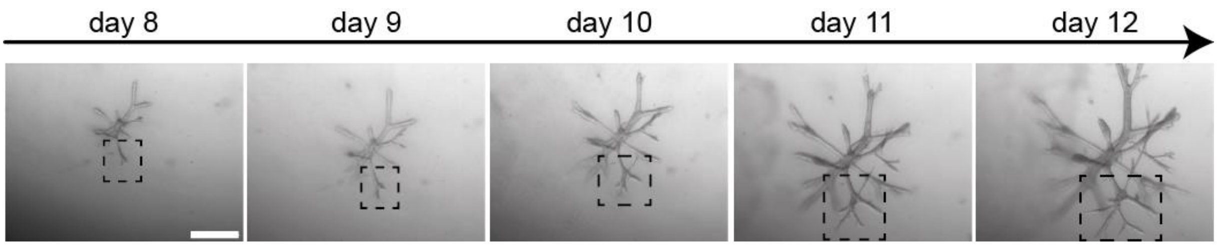

Fig. S6: Bifurcation of branch tips. Bright field images of mammary organoids taken every 24 hrs during organoid development. Boxes depict bifurcation events. Scale bar, 500 µm. Source data are provided as a Source Data file.

Donor	Age (years)
M20	67
M21	61
M23	65
M26	34
M28	38

Table S1: Reduction mammoplasty donors. The parity of the donors varied between 1 and 2.

Epitope [Clone]	Conjugation	Host	Dilution	Catalog number	Supplier
alpha smooth muscle actin	-	Rabbit	1/100	ab5964	Abcam, Cambridge, UK
E-cadherin [HECD1]	-	Mouse	1/100	ab1416	Abcam, Cambridge, UK
Gata3 [L50-823]	-	Mouse	1/250	CM405	Biocare Medical, Concord, US
Ki67	-	Rabbit		ab15580	Abcam, Cambridge, UK
Laminin	-	Rabbit	1/100	L9393	Sigma, Steinheim, Germany
P63 [EPR5701]	-	Rabbit	1/300	ab124762	Abcam, Cambridge, UK
Phalloidin	Atto 647	-	1/250	65906	Sigma, Steinheim, Germany
MMP9 [56-2A4]	-	Mouse	1/100	ab58803	Abcam, Cambridge, UK
Integrin α6 [GOH3]	-	Rat	1/100	sc-19622	Santa Cruz, Dallas, US

Table S2: Primary antibodies.

Host/Isotype	Species reactivity	Conjugation	Dilution	Catalog number	Supplier
Donkey/IgG	Mouse	Alexa 488	1/250	A-21202	Life Technologies, Darmstadt, Germany
Donkey/IgG	Rat	Alexa 488	1/250	A-21208	Life Technologies, Darmstadt, Germany
Donkey/IgG	Rabbit	Alexa 546	1/250	A-10040	Life Technologies, Darmstadt, Germany

Table S3: Secondary antibodies.

Epitope [Clone]	Conjugation	Host	Volume (µL) *	Catalog number	Supplier
7-AAD	-	-	2	559925	BD, Heidelberg, Germany
CD10 [HI10A]	APC	Mouse	2.5	312210	Biozol, Eching, Germany
CD31 [WM59]	PB	Mouse	0.5	303114	Biozol, Eching, Germany
CD326/EPCAM [VU-1D9]	FITC	Mouse	5	GTX79849	Biozol, Eching, Germany
CD45 [HI30]	V450	Mouse	0.5	560367	Biozol, Eching, Germany
CD49F [GOH3]	PE	rat	2.5	555736	BD, Heidelberg, Germany

* used to stain 1 x10^6 cells

Table S4: Antibodies used for flow cytometry and fluorescence activated cell sorting.

Experiment		P-value
Length distribution (Fig. 1C)	Establishment– Branch elongation	4.0632e-12
	Branch elongation - Alveologenesis	4.9124e-30
	Establishment - Alveologenesis	2.3018e-19
Collagen intensity (Fig. 4D)	Side- Front	1.4157e-09
	Farfield - Front	1.4941e-06
	Farfield - Side	1.4144e-09
Comparison luminal basal (Fig. S1H)	Luminal - Basal	3.4660e-14
Tip exchange (Fig. S2C)	Length	0.0418
(Fig. S2D)	Width	0.0011
Branch length (Fig. S3F)	Elongation phase	0.0132
	Alveologenesis phase	0.0012
Branch width (Fig. S3G)	Elongation phase	6.6244e-12
	Alveologenesis phase	9.2111e-17
Fiber alignment (Fig. S4C)	Front - Side	8.2327e-04
	Front - Control	6.2846e-06
	Side - Control	1.0284e-04
	Front – Cytochalasin D	0.3787
	Front - HECD1	7.0685e-04

Table S5: Statistics analysis.
