# Supplementary material for: Mechanical plasticity of collagen directs branch elongation in human mammary gland organoids
Source: Nat Commun. 2021 May 12;12:2759. doi: 10.1038/s41467-021-22988-2 (PMC8115695; doi:10.1038/s41467-021-22988-2)
Supplement: Supplementary file 3 — Description of Additional Supplementary Files [file 41467_2021_22988_MOESM3_ESM.pdf]

Description of Additional Supplementary Files

File Name: Supplementary Movie 1
Description: **Organoid growth** During the branch elongation phase beginning from day 7 the typical deformation field can be observed by tracking fluorescent beads embedded inside the collagen gel. Nuclei labelling reveals highly dynamic cell motion during the organoid growth. Cells within the organoids are labelled using sirDNA. Time between the frames was set to 10 mins.

File Name: Supplementary Movie 2
Description: **Y-27632 treatment** Organoid behavior upon treatment with 10 µM Y-27632 at day 10 shows the formation of filopodia-like structures. Time between the frames was set to 10 mins.

File Name: Supplementary Movie 3
Description: **Protrusion dynamics** during invasion The actin network is steadily remodeled during the invasion process and forms invasive protrusions. In order to image the actin network, cells were transfected with LifeAct. Time between the frames was set to 15 mins.

File Name: Supplementary Movie 4
Description: **Tip cell exchange** Stalk cells occasionally replace leading cells. Cells are labelled with sirDNA. Time between the frames was set to 10 mins

File Name: Supplementary Movie 5
Description: **Cytochalasin D treatment** Relaxation field upon treatment with 10 µM Cytochalasin D shows counter balance between tensile forces of the branch and restoring force of the collagen. Time between the frames is set to 10 min.

File Name: Supplementary Movie 6
Description: **Laser ablation** Relaxation of a branch after laser ablation of the collagen in close proximity to the tip. Time between the frames is set to 1 sec.

File Name: Supplementary Movie 7
Description: **HECD1 treatment** Organoid growth upon treatment with HECD1 at a dilution of 1:50. Cells were labelled using sirDNA. Time between the frames was set to 10 mins.

File Name: Supplementary Movie 8
Description: **Collagen invasion** Fluorescent collagen reveals that leading cells do not continuously attach to the same collagen fibers but change their attachment sites over time. Cells were labelled using sirDNA. Time between the frames was set to 10 mins

File Name: Supplementary Movie 9
Description: **High-resolution imaging of the collagen cage** 3D visualization of the collagen cage around an organoid using a deconvolution algorithm. The color code refers to the height of the slice.

File Name: Supplementary Movie 10
Description: **Marimastat treatment** Organoid growth upon treatment with 10µM Marimastat shows stop of branch elongation and thickening of the branches. Cells were labelled using sirDNA. Time between the frames was set to 20 mins.

File Name: Supplementary Movie 11
Description: **Cage formation** Collagen gets pushed to the sides by the tip cell, leading to the accumulation of collagen at the sides, highlighted by the use of fluorescent collagen. Cells were labelled using sirDNA. Time between the frames was set to 10 mins.
