# Supplementary material for: Mechanical plasticity of collagen directs branch elongation in human mammary gland organoids
Source: Nat Commun. 2021 May 12;12:2759. doi: 10.1038/s41467-021-22988-2 (PMC8115695; doi:10.1038/s41467-021-22988-2)
Supplement: Supplementary file 15 — Reporting Summary [file 41467_2021_22988_MOESM15_ESM.pdf]

nature research

Corresponding author(s): Prof. Dr. Andreas Bausch

Last updated by author(s): Apr 6, 2021

Reporting Summary

Nature Research wishes to improve the reproducibility of the work that we publish. This form provides structure for consistency and transparency in reporting. For further information on Nature Research policies, see our Editorial Policies and the Editorial Policy Checklist.

Statistics

For all statistical analyses, confirm that the following items are present in the figure legend, table legend, main text, or Methods section.

n/a	Confirmed	
☐	☒	The exact sample size (n) for each experimental group/condition, given as a discrete number and unit of measurement
☐	☒	A statement on whether measurements were taken from distinct samples or whether the same sample was measured repeatedly
☐	☒	The statistical test(s) used AND whether they are one- or two-sided<br>*Only common tests should be described solely by name; describe more complex techniques in the Methods section.*
☒	☐	A description of all covariates tested
☒	☐	A description of any assumptions or corrections, such as tests of normality and adjustment for multiple comparisons
☐	☒	A full description of the statistical parameters including central tendency (e.g. means) or other basic estimates (e.g. regression coefficient) AND variation (e.g. standard deviation) or associated estimates of uncertainty (e.g. confidence intervals)
☐	☒	For null hypothesis testing, the test statistic (e.g. F, t, r) with confidence intervals, effect sizes, degrees of freedom and P value noted<br>*Give P values as exact values whenever suitable.*
☒	☐	For Bayesian analysis, information on the choice of priors and Markov chain Monte Carlo settings
☒	☐	For hierarchical and complex designs, identification of the appropriate level for tests and full reporting of outcomes
☒	☐	Estimates of effect sizes (e.g. Cohen's d, Pearson's r), indicating how they were calculated

Our web collection on statistics for biologists contains articles on many of the points above.

Software and code

Policy information about availability of computer code

Data collection	Flow cytometry data were analysed using FlowJo V10. Live-cell imaging was done using Leica Application Suite X Version 3.57. The high-resolution cage analysis was done using the implemented deconvolution algorithm Lightning.
Data analysis	For immunofluorescence and basic image analysis ImageJ 1.52p was used. Bead tracking and data analysis was done using Matlab R2019.

For manuscripts utilizing custom algorithms or software that are central to the research but not yet described in published literature, software must be made available to editors and reviewers. We strongly encourage code deposition in a community repository (e.g. GitHub). See the Nature Research guidelines for submitting code & software for further information.

Data

Policy information about availability of data

All manuscripts must include a data availability statement. This statement should provide the following information, where applicable:

- Accession codes, unique identifiers, or web links for publicly available datasets
- A list of figures that have associated raw data
- A description of any restrictions on data availability

Microscopy data that support the findings of this study have been deposited in Zenodo with the identifier 10.5281/zenodo.4590475 [http://dx.doi.org/10.5281/zenodo.4590475]. All other relevant data supporting the key findings of this study are available within the article and its Supplementary Information files or from the corresponding author upon reasonable request. Source data are provided with this paper.

Field-specific reporting

Please select the one below that is the best fit for your research. If you are not sure, read the appropriate sections before making your selection.

- [x] Life sciences
- [ ] Behavioural & social sciences
- [ ] Ecological, evolutionary & environmental sciences

For a reference copy of the document with all sections, see nature.com/documents/nr-reporting-summary-flat.pdf

Life sciences study design

All studies must disclose on these points even when the disclosure is negative.

Sample size	The number of biological and technical replicates were chosen according to previously published experimental designs (Linnemann et al,2015) and are specified for each experiment in the figure legends.
Data exclusions	No data was excluded.
Replication	All experiments were repeated successfully for at least three different donors and for at least five different organoids.
Randomization	Donor samples were picked according to age and parity in order to provide a broad biological variation, as seen in Table S1. However, between the different donors no differences in organoid morphology, cell migration or contractile behaviour was observable. Organoids were chosen according to days in cultivation, developmental stage, size and complexity to enhance comparability. In particular, only organoids after day 7 of cultivation with a diameter larger than 200 μm with at least primary side branches were analysed regarding their branch elongation and cage formation.
Blinding	Blinding was not necessary as data interpretation is based on quantitative measurements with no data being excluded.

Reporting for specific materials, systems and methods

We require information from authors about some types of materials, experimental systems and methods used in many studies. Here, indicate whether each material, system or method listed is relevant to your study. If you are not sure if a list item applies to your research, read the appropriate section before selecting a response.

Materials & experimental systems

n/a	Involved in the study	
[ ]	[x]	Antibodies
[ ]	[x]	Eukaryotic cell lines
[x]	[ ]	Palaeontology and archaeology
[x]	[ ]	Animals and other organisms
[ ]	[x]	Human research participants
[x]	[ ]	Clinical data
[x]	[ ]	Dual use research of concern

Methods

n/a	Involved in the study	
[x]	[ ]	ChIP-seq
[ ]	[x]	Flow cytometry
[x]	[ ]	MRI-based neuroimaging

Antibodies

Antibodies used

alpha smooth muscle actin - Rabbit 1/100 ab5964 Abcam, Cambridge, UK
E-cadherin [HECD1] - Mouse 1/100 ab1416 Abcam, Cambridge, UK
Gata3 [L50-823] - Mouse 1/250 CM405 Biocare Medical, Concord, US
Ki67 - Rabbit ab15580 Abcam, Cambridge, UK
Laminin - Rabbit 1/100 L9393 Sigma, Steinheim, Germany
P63 [EPR5701] - Rabbit 1/300 ab124762 Abcam, Cambridge, UK
Phalloidin Atto 647 - 1/250 65906 Sigma, Steinheim, Germany
MMP9 [56-2A4] - Mouse 1/100 ab58803 Abcam, Cambridge, UK
Integrin α6 [GOH3] - Rat 1/100 sc-19622 Santa Cruz, Dallas, US

Donkey/IgG Mouse Alexa 488 1/250 A-21202 Life Technologies, Darmstadt, Germany
Donkey/IgG Rat Alexa 488 1/250 A-21208 Life Technologies, Darmstadt, Germany
Donkey/IgG Rabbit Alexa 546 1/250 A-10040 Life Technologies, Darmstadt, Germany

7-AAD - - 2 559925 BD, Heidelberg, Germany
CD10 [HI10A] APC Mouse 2.5 312210 Biozol, Eching, Germany
CD31 [WM59] PB Mouse 0.5 303114 Biozol, Eching, Germany
CD326/EPCAM [VU-1D9] FITC Mouse 5 GTX79849 Biozol, Eching, Germany

CD45 [HI30] V450 Mouse 0.5 560367 Biozol, Eching, Germany
CD49F [GOH3] PE rat 2.5 555736 BD, Heidelberg, Germany

Validation

Primary antibody against alpha smooth muscle actin (Abcam, ab5964) has been validated to react with human samples as shown by immunohistochemistry analysis of human stomach tissue provided on the manufacture website. Primary antibody against alpha smooth muscle actin (Abcam, ab5964) has been validated to work for immunofluorescence analysis as shown by labelling of A-673 cells (human muscle Ewing's Sarcoma cell line) provided on the manufacture website.
Primary antibody against E-cadherin (Abcam, ab1416) has been validated to react with human samples as shown by immunohistochemistry analysis of human breast cancer tissue provided on the manufacture website.
Primary antibody against Ki67 (Abcam, ab15580) has been validated to react with human samples as shown by immunohistochemistry analysis of human spleen formalin fixed paraffin embedded tissue section provided on the manufacture website. Primary antibody against Ki67 (Abcam, ab15580) has been validated to work for immunofluorescence analysis as shown by labelling of HeLa cells provided on the manufacture website.
Primary antibody against p63 (Abcam, ab124762) has been validated to react with human samples as shown by immunohistochemistry analysis of human breast tissue sections provided on the manufacture website. Primary antibody against p63 (Abcam, ab124762) has been validated to work for immunofluorescence analysis as shown by labelling of human corneal limbal epithelial cells (primary culture) provided on the manufacture website.
Primary antibody against MMP9 (Abcam, ab58803) has been validated to react with human samples and to work for immunofluorescence analysis as shown by labelling of the human Panc1 cell line provided on the manufacture website.
Primary antibodies against Gata3 (Biocare Medical, CM405), Laminin (Sigma, L9393) and Integrin α6 (Santa Cruz, sc-19622) have been validated to react with human samples and to work for immunofluorescence analysis as shown by labelling human mammary epithelial cells as previously described (Linnemann et al., 2015).
Antibodies used for flow cytometry have been validated to react with human samples and to work for flow cytometry analysis as shown by labelling and flow cytometry analysis of primary human mammary cells as previously described (Linnemann et al., 2015).

Eukaryotic cell lines

Policy information about cell lines

Cell line source(s)

HEK293T high performance cells were bought from ATCC.

Authentication

None of the cell lines were authenticated.

Mycoplasma contamination

Cells were not tested for mycoplasma contamination.

Commonly misidentified lines
(See ICLAC register)

No commonly misidentified cell lines were used in this study.

Human research participants

Policy information about studies involving human research participants

Population characteristics

Women undergoing aesthetic reduction mammoplasties were healthy, between 38 and 67 years old and had undergone either one or two pregnancies. Donor specific information concerning age and parity is provided within the manuscript.

Recruitment

Participants were not recruited. Breast tissue samples were collected from patients undergoing aesthetic reduction mammoplasties and sample collection was unbiased and completely randomized. Donors were of different age and parity and tissue samples were irreversible anonymized.

Ethics oversight

The study protocol was approved by the ethics committee of the Ludwig-Maximilian University, Munich, Germany (proposal 397-12)

Note that full information on the approval of the study protocol must also be provided in the manuscript.

Flow Cytometry

Plots

Confirm that:

- [x] The axis labels state the marker and fluorochrome used (e.g. CD4-FITC).
- [x] The axis scales are clearly visible. Include numbers along axes only for bottom left plot of group (a 'group' is an analysis of identical markers).
- [x] All plots are contour plots with outliers or pseudocolor plots.
- [x] A numerical value for number of cells or percentage (with statistics) is provided.

Methodology

Sample preparation	Primary human mammary cells were thawn, washed and the single cell suspension was stained for CD31-PB,CD45-, V450, CD49f-PE, EpCAM-FITC and CD10-APC. Unstained and single stained controls were added. 7AAD was added to the suspension for dead cell exclusion. Luminal progenitors (EpCAMhi/CD49fhi) and basal cells (EpCAMlow/CD49fhi/CD10+) were sorted.
Instrument	FACS Aria III
Software	FlowJo V10 software was used for post-analysis.
Cell population abundance	The sort purity was determined by re-analysis.
Gating strategy	FCS-A and SSC-A as well as FSC-W and SSC-W were used to discriminate duplets. From the 7AAD negative population we excluded hematopoietic and endothelial cells by applying markers CD31 and CD45, subsequently basal cells were determined based on their expression of CD49f and CD10.

☒ Tick this box to confirm that a figure exemplifying the gating strategy is provided in the Supplementary Information.
